# Supplementary material for: Lead binds HIF-1α contributing to depression-like behaviour through modulating mitochondria-associated astrocyte ferroptosis
Source: Commun Biol. 2025 Sep 11;8:1342. doi: 10.1038/s42003-025-08824-z (PMC12426229; doi:10.1038/s42003-025-08824-z)

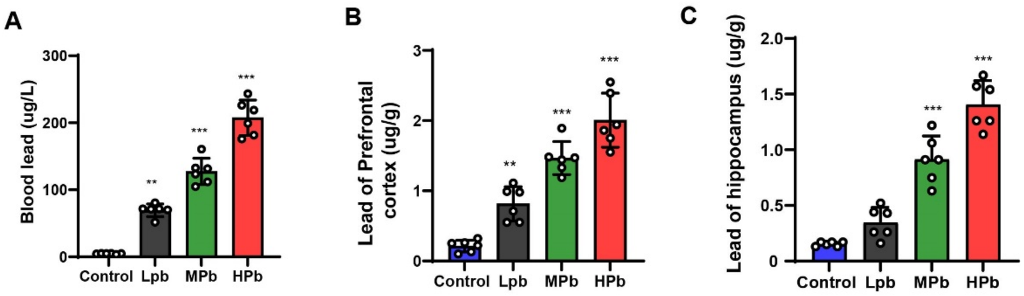

Supplementary Figure1 The concentrations of Pb^{2+} in blood and prefrontal cortex of LPb, MPb, and HPb groups.

The concentrations of Pb^{2+} in **A.** blood; **B.** prefrontal cortex; **C.** hippocampus. $^{*}P<0.05$ vs Control; $^{**}P<0.01$ vs Control; $^{***}P<0.001$ vs Control; Comparisons are made using ANOVA, followed by Tukey's multiple comparison test.

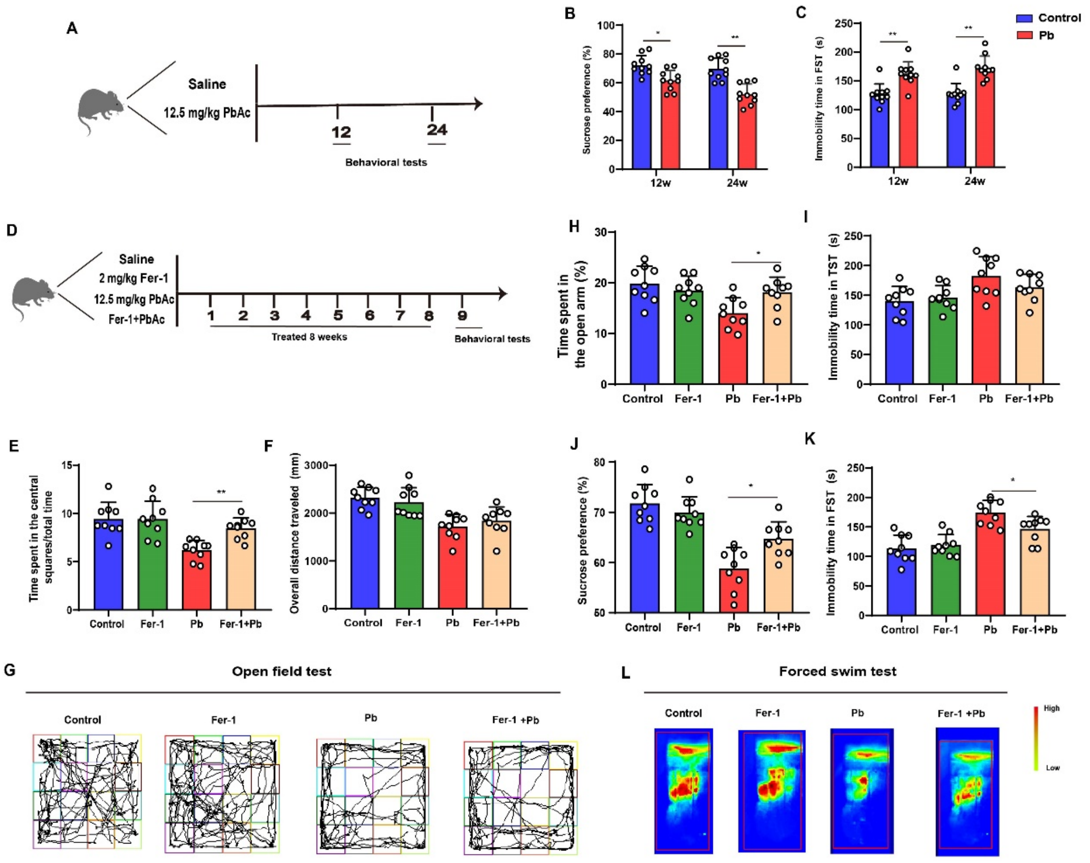

Supplementary Figure2 Fer-1 inhibited anxiety- and depression-like behavior of mice following Pb exposure.

A. Flow chart of animal treatment and behavioral test. SPF male C57BL/6J mice were divided

into control, Pb groups; The **B.** sucrose preference and **C.** immobility time of FST in Control, Pb (n=10); **D.** Flow chart of animal treatment and behavioral test. SPF male C57BL/6J mice were divided into control, Fer-1, Pb and Fer-1+Pb groups; The **E.** Time spent of the central squares, **F.** Overall distance travelled**, G.** Moving trajectory in OFT**, H.** time spent of the open arm of EPM, **I.** immobility time of TST, **J.** sucrose preference, **K.** immobility time, and **L.** activity of FST in control, Fer-1, Pb and Fer-1+Pb groups (n=10). $^{*}P$<0.05 vs indicated group; $^{**}P$<0.01 vs indicated group; $^{***}P$<0.001 vs indicated group. Comparisons are made using ANOVA, followed by Tukey's multiple comparison test.

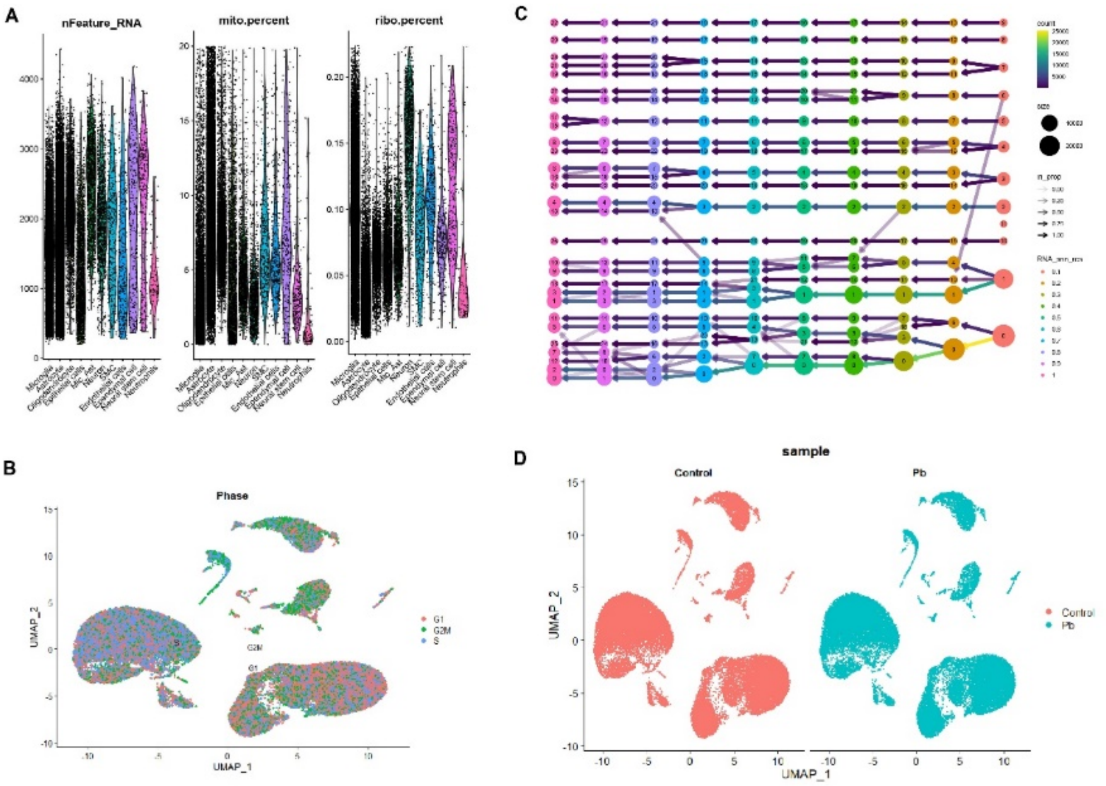

Supplementary Figure3 The standardization analysis of scRNA-seq in mice brain following Pb exposure.

A. The nFeature_RNA, mito.percent and ribo.percent in all cell type based on ScRNA-seq; **B.** The cell cycle effects visualized at UMAP plot in in all cell type; **C.** The clustering tree of all cluster based on ScRNA-seq; **D.** The UMAP plot in different groups.

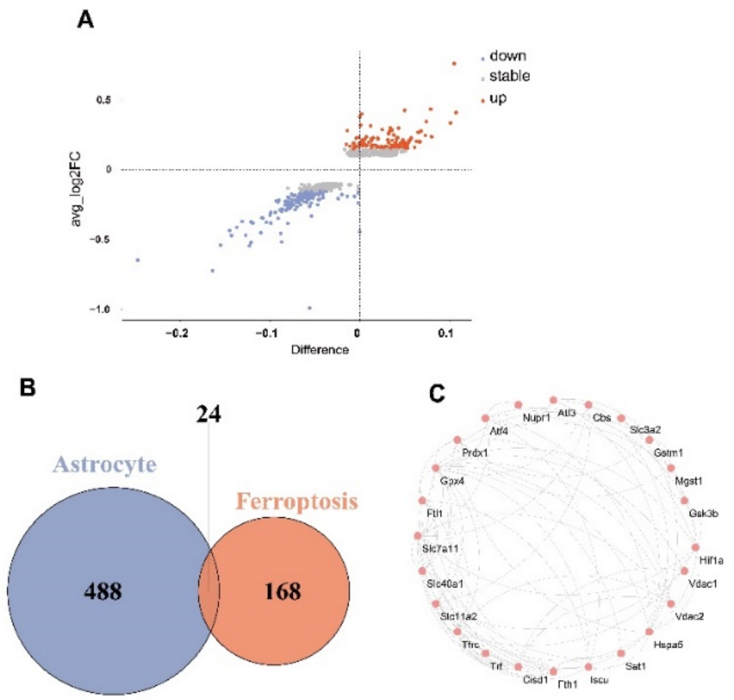

Supplementary Figure4 The differential gene expression of ferroptosis related in astrocyte following Pb exposure

A. Volcano plot of mRNA profiling in astrocyte following Pb exposure; **B.** Venn diagram of ferroptosis-related DEGs between the control and Pb groups in astrocyte; **C.** The PPI network of ferroptosis-related DEGs in astrocyte.

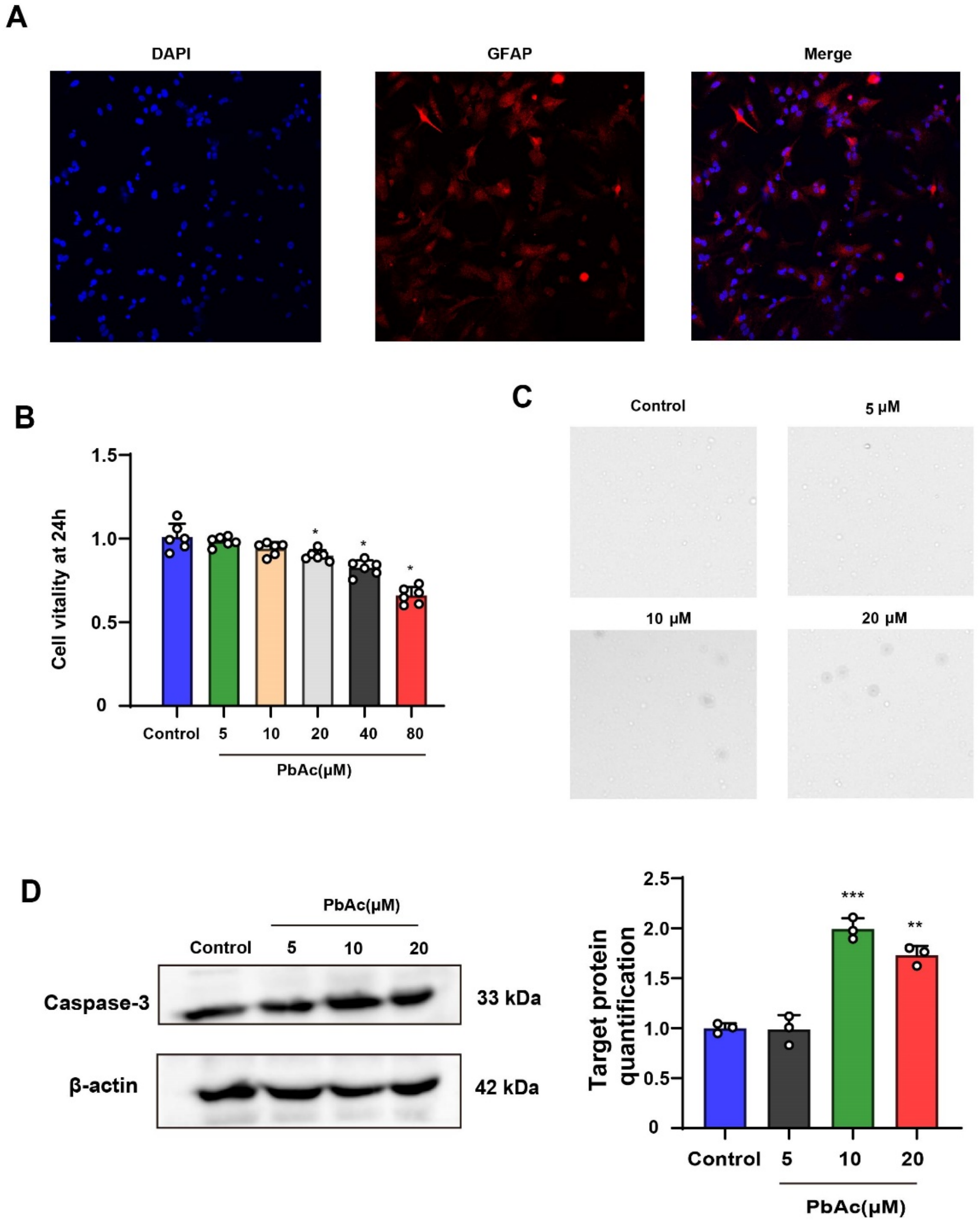

Supplementary Figure5 Pb induced the astrocyte death in dose–response relationship and the protein of apoptosis in astrocyte.

A. The Immunofluorescence image of GFAP protein in primary culture astrocyte; **B.** The vitality of astrocyte in astrocyte following Pb exposure (n=6); **C.** The morphology of the astrocyte was observed and stained with trypan blue staining in astrocyte following Pb exposure; **D.** Gel electrophoresis plot and semi-quantification of Caspase-3 in different group. $^{*}P<0.05$ vs Control; $^{**}P<0.01$ vs Control; $^{***}P<0.001$ vs Control. Comparisons are made with ANOVA, followed by Tukey's multiple comparison tests.

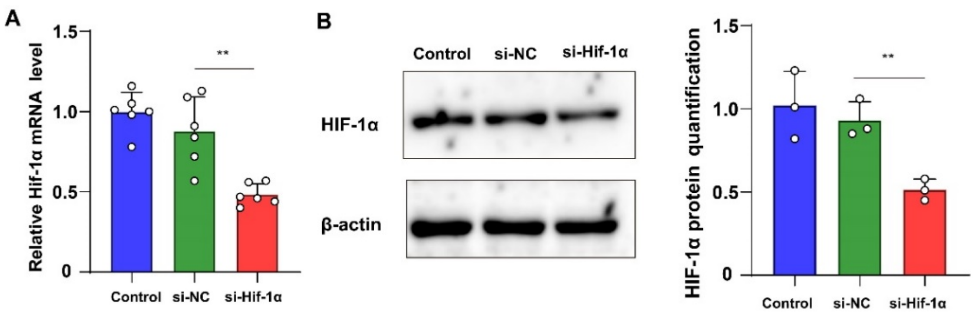

Supplementary Figure6 The mRNA and protein expression of HIF-1α in astrocyte with si-*Hif-1α*

**P*<0.05 vs Control; ***P*<0.01 vs Control; ****P*<0.001 vs Control. Comparisons are made using ANOVA, followed by Tukey's multiple comparison test.

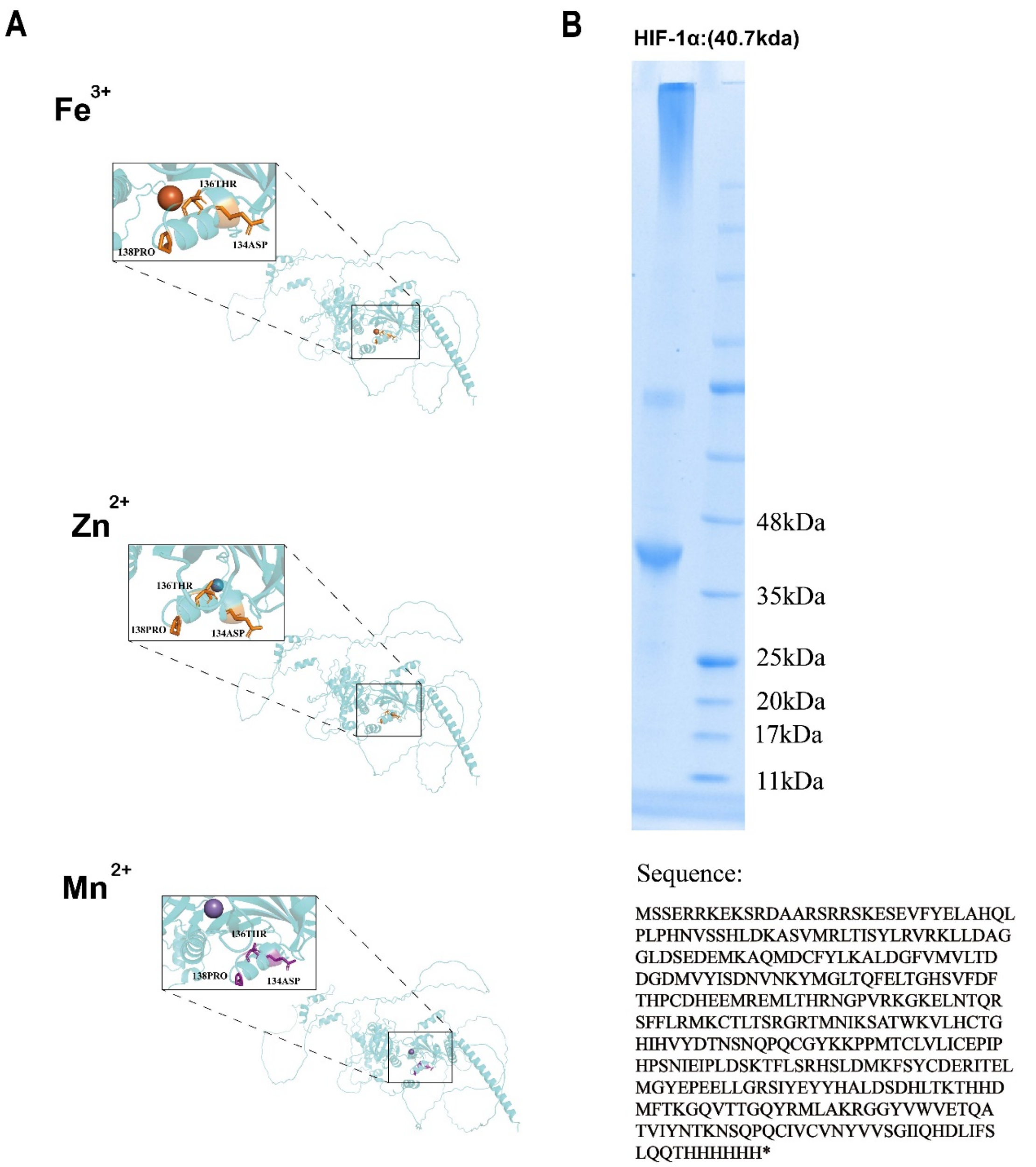

Supplementary Figure7 Molecular docking model illustrating the binding of metal to HIF-1α and the SDS-polyacrylamide gel preparation for HIF-1α protein. A. Molecular docking model illustrating the binding of metal to HIF-1α. B. The SDS-polyacrylamide gel preparation for HIF-1α protein and the Sequence of HIF-1α of protein domain

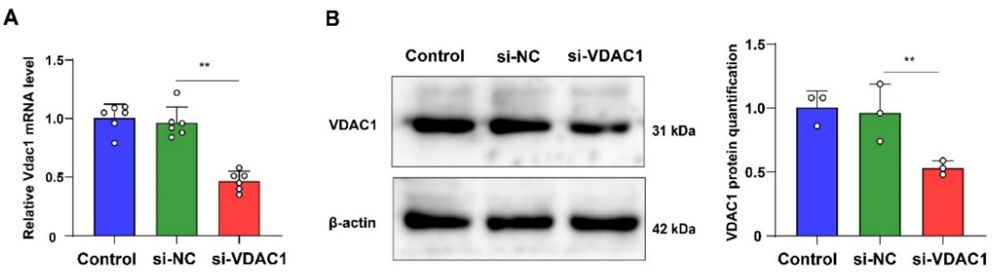

Supplementary Figure8 The mRNA and protein expression of VDAC1 in astrocyte with si-*Vdac1*

$^{*}P$<0.05 vs Control; $^{**}P$<0.01 vs Control; $^{***}P$<0.001 vs Control. Comparisons are made using ANOVA, followed by Tukey's multiple comparison test.

Supplemental Table 1 The primer sequences used for real-time PCR

Gene	Primer sequences（5'—3'）	
	Forward primer	Reverse primer
Vdac1	AGAAACGGACCTGCTCTTCCCA	CCTCTGTCTCAATGCCACCAAC
Hif-1α	TGGGGCAGTCAATGGATGAG	AAATAGACTGCTGAGCCACC
β-actin	GGAGATTACTGCCCTGGCTCCTA	GACTCATCGTACTCCTGCTTGCTG

Figure exemplifying the gating strategy

A

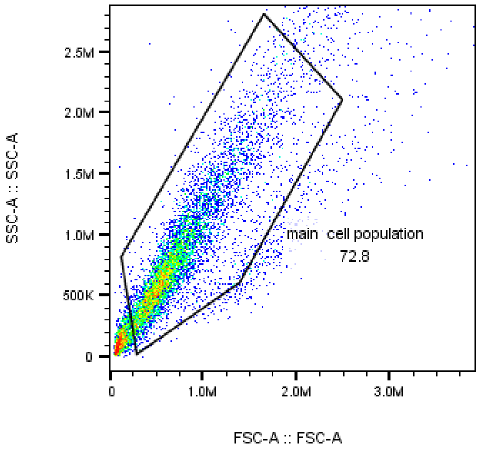

B

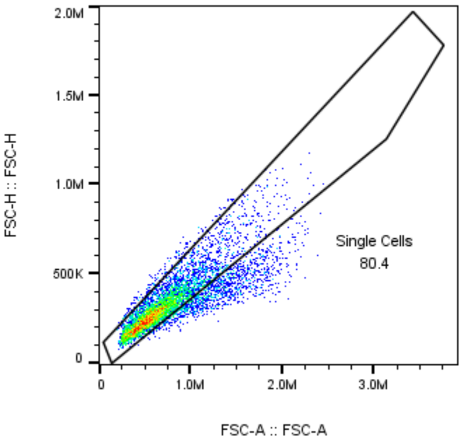

C

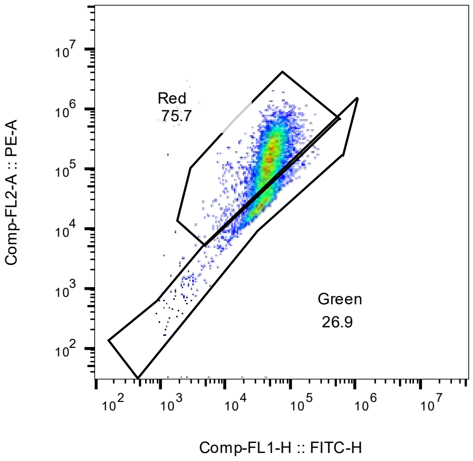

C11-BODIPY and mtSOX analysis: First, perform quality control analysis on the cells with FSC-A as the X-axis and SSC A as the Y-axis to remove cell debris. Then, set singlet gates with FSC-H as the Y-axis and FSC-A as the X-axis, select the diagonal cell population, remove adherent cells, and determine single cells for analysis (A-B).

JC-1 analysis: The positive in the PE channel and the negative in the FITC channel was selected as the gating strategy (C).

Uncropped gels for western blots in Figures

Figure 1:

Fig1 M,N

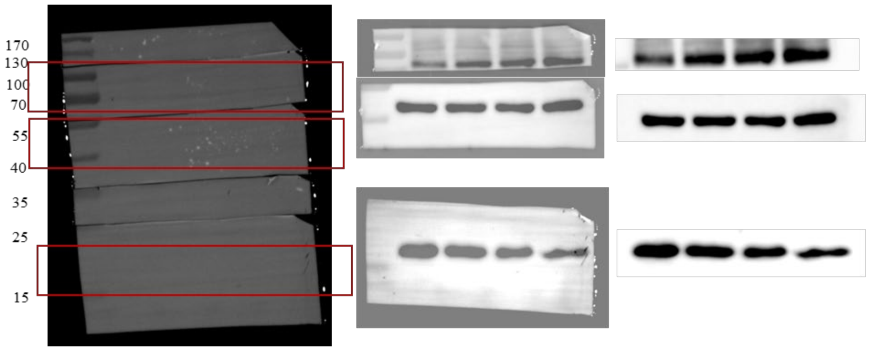

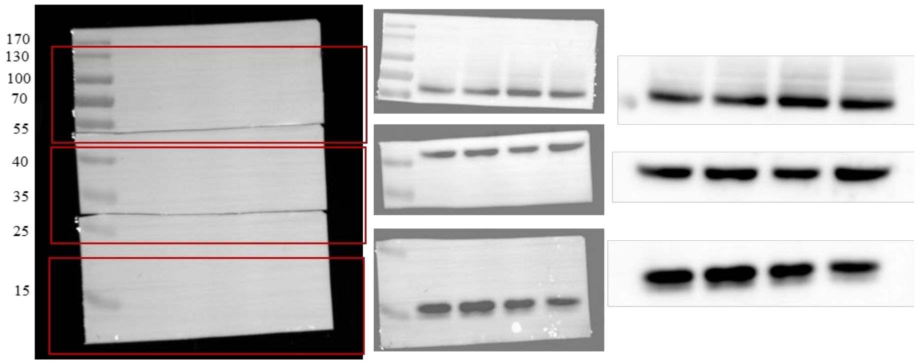

Figure 4:

Fig4 J

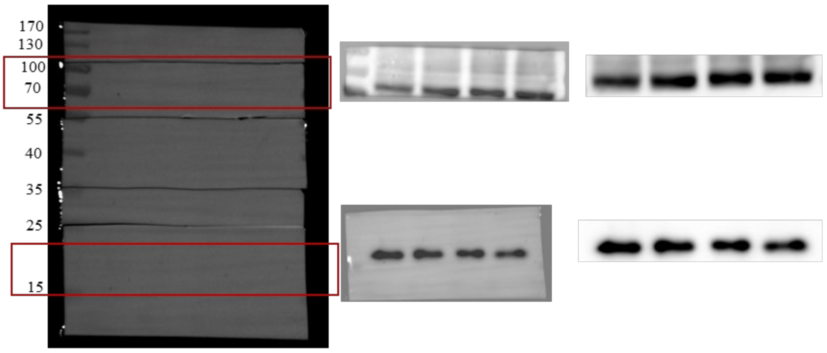

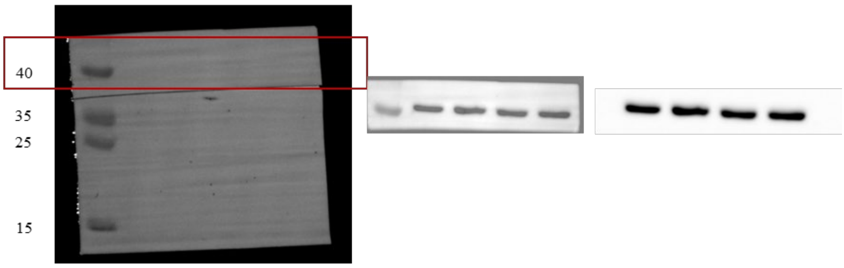

Figure 5:

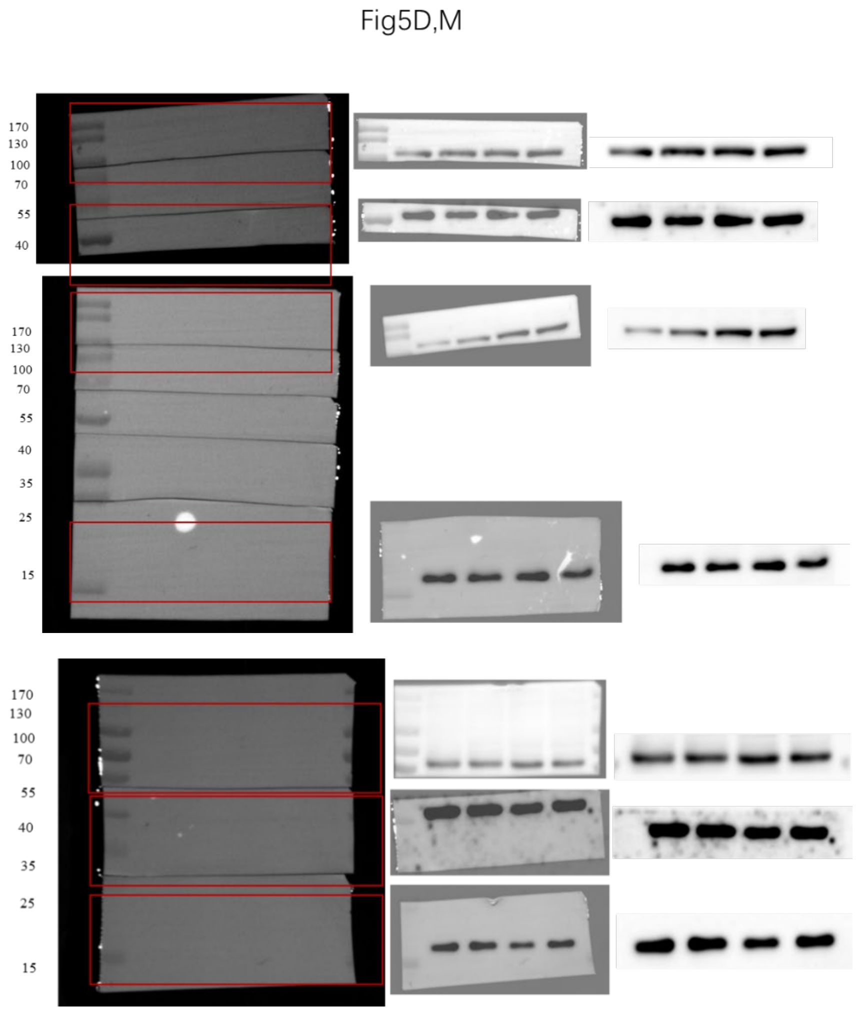

Figure 7:

Fig7 E,H

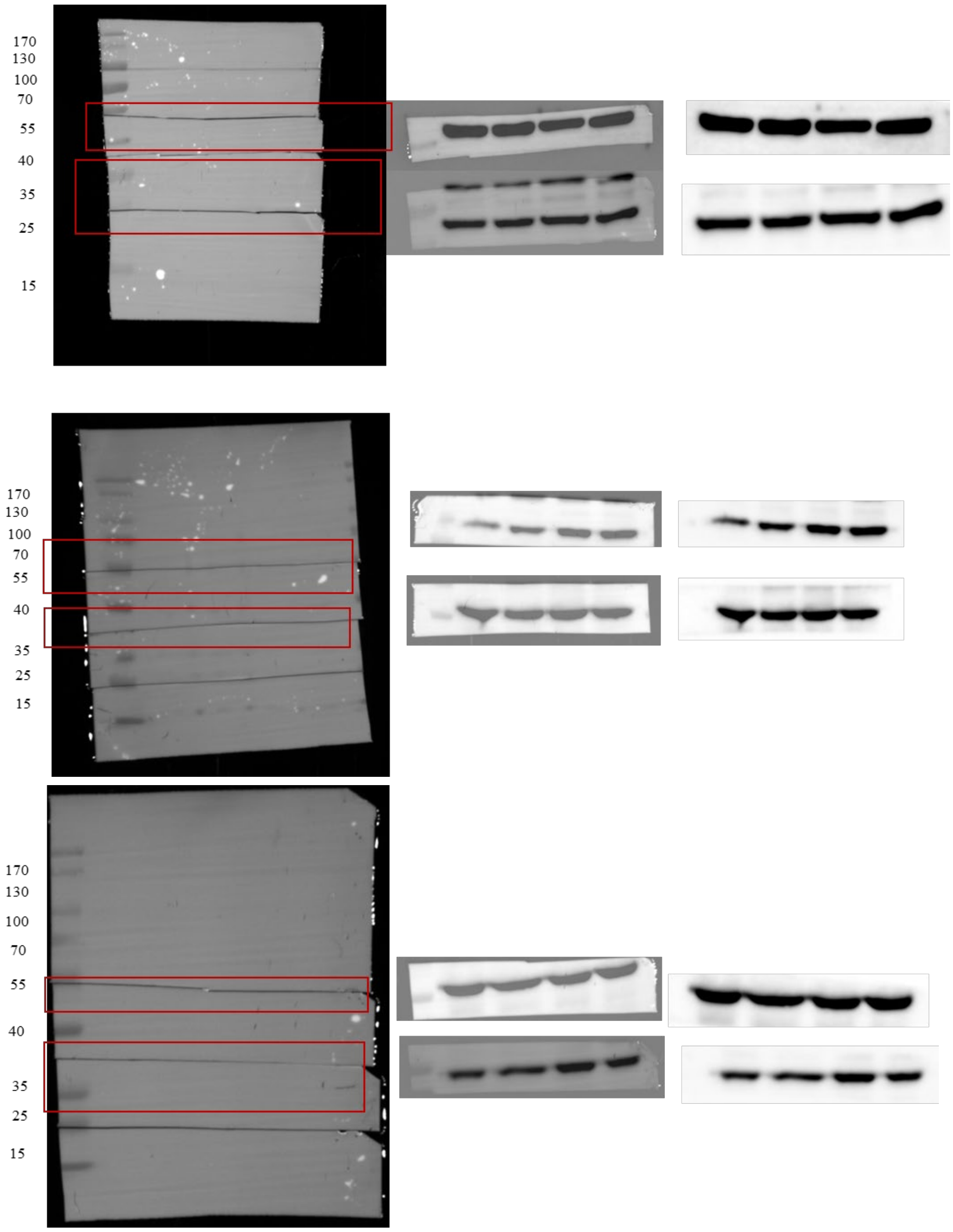

Figure 9:

Fig 9 I

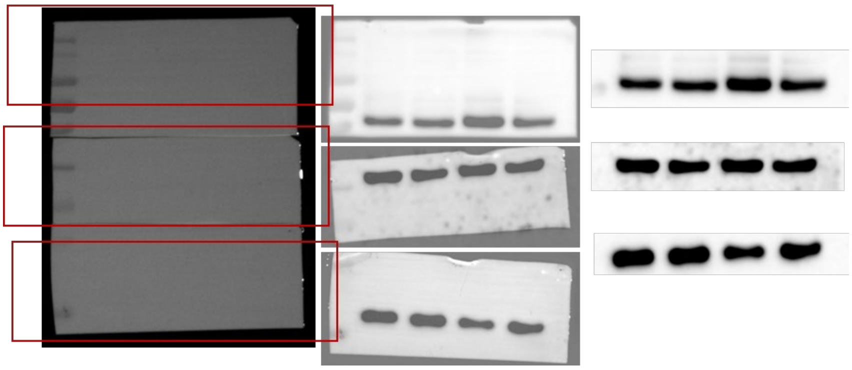

Supplementary Figure5:

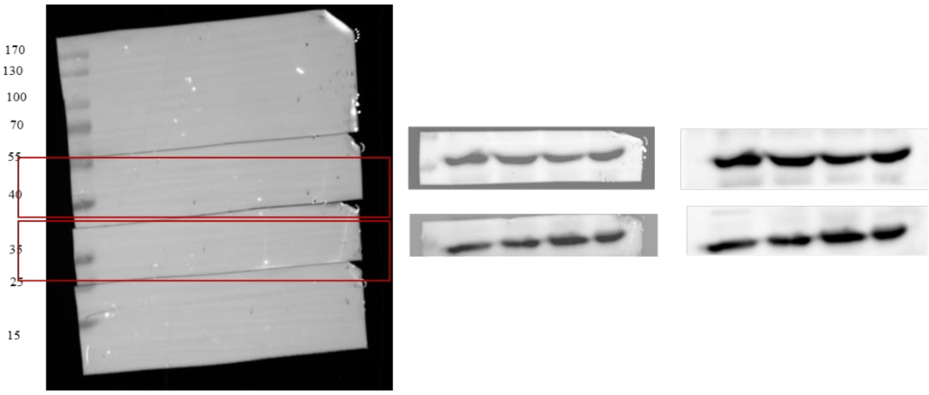

Supplementary Figure6:

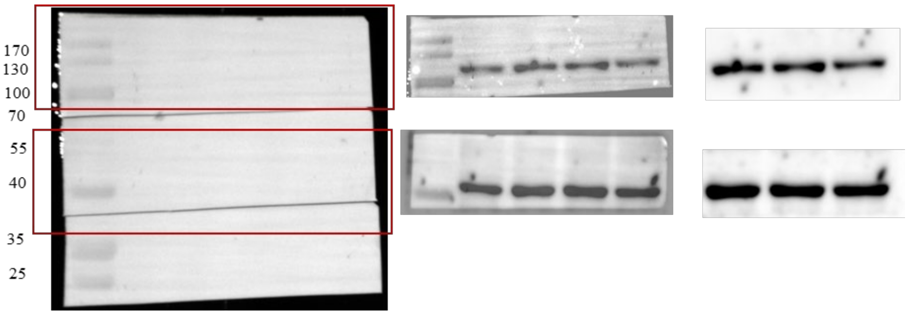

Supplementary Figure7:

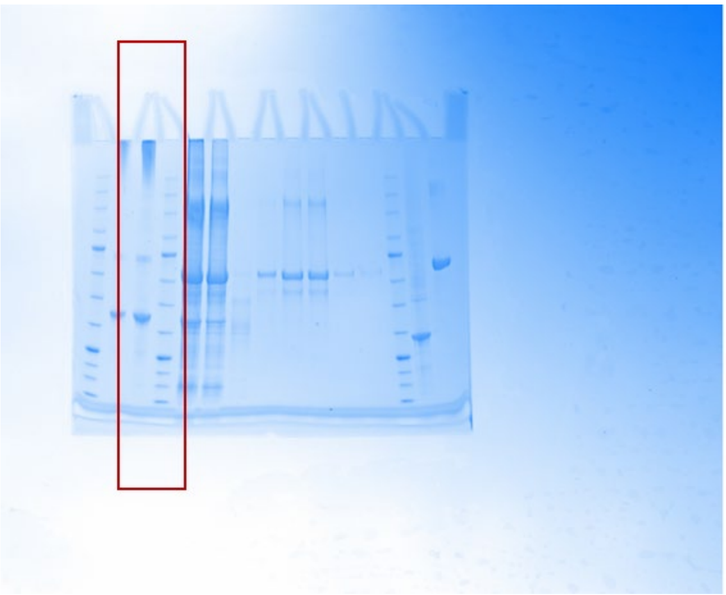

Supplementary Figure8:

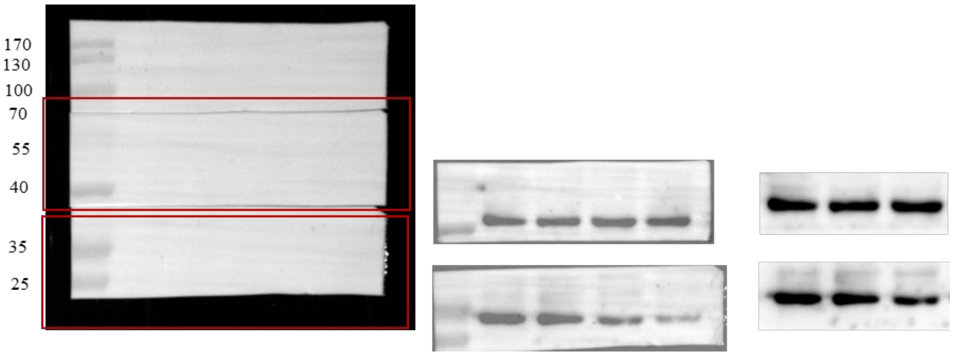

Supplement: Supplementary file 2 — Supplementary materials [file 42003_2025_8824_MOESM2_ESM.pdf]
