# Supplementary material for: Lead binds HIF-1α contributing to depression-like behaviour through modulating mitochondria-associated astrocyte ferroptosis
Source: Commun Biol. 2025 Sep 11;8:1342. doi: 10.1038/s42003-025-08824-z (PMC12426229; doi:10.1038/s42003-025-08824-z)
Supplement: Supplementary file 5 — Reporting Summary [file 42003_2025_8824_MOESM5_ESM.pdf]

nature portfolio

Corresponding author(s): Yan-Shu Zhang

Last updated by author(s): Jul 9, 2025

Reporting Summary

Nature Portfolio wishes to improve the reproducibility of the work that we publish. This form provides structure for consistency and transparency in reporting. For further information on Nature Portfolio policies, see our Editorial Policies and the Editorial Policy Checklist.

Statistics

For all statistical analyses, confirm that the following items are present in the figure legend, table legend, main text, or Methods section.

n/a	Confirmed	
☐	☒	The exact sample size (n) for each experimental group/condition, given as a discrete number and unit of measurement
☐	☒	A statement on whether measurements were taken from distinct samples or whether the same sample was measured repeatedly
☐	☒	The statistical test(s) used AND whether they are one- or two-sided<br>*Only common tests should be described solely by name; describe more complex techniques in the Methods section.*
☒	☐	A description of all covariates tested
☐	☒	A description of any assumptions or corrections, such as tests of normality and adjustment for multiple comparisons
☐	☒	A full description of the statistical parameters including central tendency (e.g. means) or other basic estimates (e.g. regression coefficient) AND variation (e.g. standard deviation) or associated estimates of uncertainty (e.g. confidence intervals)
☐	☒	For null hypothesis testing, the test statistic (e.g. F, t, r) with confidence intervals, effect sizes, degrees of freedom and P value noted<br>*Give P values as exact values whenever suitable.*
☒	☐	For Bayesian analysis, information on the choice of priors and Markov chain Monte Carlo settings
☒	☐	For hierarchical and complex designs, identification of the appropriate level for tests and full reporting of outcomes
☒	☐	Estimates of effect sizes (e.g. Cohen's d, Pearson's r), indicating how they were calculated

Our web collection on statistics for biologists contains articles on many of the points above.

Software and code

Policy information about availability of computer code

Data collection	The behavioral video tracking system were collected from SMART3.0 (Xinruan, Shang Hai); sc-RNAseq-Raw data were sequencing from Bohao Shang Hai);Clinical data were obtain from Genotype-Tissue Expression
Data analysis	All packages for data analysis used in this study were open source in R software (version 4.2.3), Online tools analyzed the Clinical data (https://jingle.shinyapps.io/TF_Target_Finder/)

For manuscripts utilizing custom algorithms or software that are central to the research but not yet described in published literature, software must be made available to editors and reviewers. We strongly encourage code deposition in a community repository (e.g. GitHub). See the Nature Portfolio guidelines for submitting code & software for further information.

Data

Policy information about availability of data

All manuscripts must include a data availability statement. This statement should provide the following information, where applicable:

- Accession codes, unique identifiers, or web links for publicly available datasets
- A description of any restrictions on data availability
- For clinical datasets or third party data, please ensure that the statement adheres to our policy

1,152 human autopsy samples of brain sequence were obtained from the Genotype-Tissue Expression

Research involving human participants, their data, or biological material

Policy information about studies with human participants or human data. See also policy information about sex, gender (identity/presentation), and sexual orientation and race, ethnicity and racism.

Reporting on sex and gender	Not applicable.
Reporting on race, ethnicity, or other socially relevant groupings	Not applicable.
Population characteristics	Not applicable.
Recruitment	Not applicable.
Ethics oversight	Not applicable.

Note that full information on the approval of the study protocol must also be provided in the manuscript.

Field-specific reporting

Please select the one below that is the best fit for your research. If you are not sure, read the appropriate sections before making your selection.

☒ Life sciences ☐ Behavioural & social sciences ☐ Ecological, evolutionary & environmental sciences

For a reference copy of the document with all sections, see nature.com/documents/nr-reporting-summary-flat.pdf

Life sciences study design

All studies must disclose on these points even when the disclosure is negative.

Sample size	The number of mice in the experiment was determined based on general principles of animal research and previous literature reports.
Data exclusions	The animals were excluded if they suffered accidental death or injured due to fighting.
Replication	To validate the reproducibility ofthe reults, we conducted repeated experiments in at least three batches of animals. All experiments yieldedconsistent results, indicating that our findings are highly reproducible
Randomization	In this study, animals are randomly assigned to the control and LPb, MPb, HPb, Fer-1, Fer+Pb group using a computer-generated random number table toensure group balance and reduce selection bias.
Blinding	A double-blind method is used in behavioral experiments.

Reporting for specific materials, systems and methods

We require information from authors about some types of materials, experimental systems and methods used in many studies. Here, indicate whether each material, system or method listed is relevant to your study. If you are not sure if a list item applies to your research, read the appropriate section before selecting a response.

Materials & experimental systems

n/a	Involved in the study	
☐	☒	Antibodies
☐	☒	Eukaryotic cell lines
☒	☐	Palaeontology and archaeology
☐	☒	Animals and other organisms
☒	☐	Clinical data
☒	☐	Dual use research of concern
☒	☐	Plants

Methods

n/a	Involved in the study	
☒	☐	ChIP-seq
☐	☒	Flow cytometry
☒	☐	MRI-based neuroimaging

Antibodies

Antibodies used	GPX4 (1:1000,30388-1-AP),PTGS2(1:500,27308-1-AP),HIF-1α (1:4000, 20960-1-AP),HISTONE H3 (1:6000,13754-1-AP), VDAC1 (1:2000,55259-1-AP) rabbit (Proteintech, China); β-actin (1:3000,66009-1-Ig) VDAC2 (1:1000,66388-1-Ig) mouse (Proteintech,

China).

Validation

GPX4 :https://www.ptgcn.com/products/GPX4-Antibody-30388-1-AP.htm
PTGS2:https://www.ptgcn.com/products/COX2--Cyclooxygenase-2-Antibody-27308-1-AP.htm
HIF-1α:https://www.ptgcn.com/products/HIF1A-Antibody-20960-1-AP.htm
HISTONE H3 :https://www.ptgcn.com/products/H3F3A-Antibody-13754-1-AP.htm
VDAC2 :https://www.ptgcn.com/products/VDAC2-Antibody-66388-1-Ig.htm
VDAC1 :https://www.ptgcn.com/products/VDAC1-Antibody-55259-1-AP.htm
β-actin:https://www.ptgcn.com/products/Pan-Actin-Antibody-66009-1-Ig.htm

Eukaryotic cell lines

Policy information about cell lines and Sex and Gender in Research

Cell line source(s)	C8-D1A Mouse astrocytes
Authentication	Cell lines were purchased directly from Pricella
Mycoplasma contamination	All cell lines were tested negative for mycoplasma, Certified by the company.
Commonly misidentified lines (See ICLAC register)	N/A

Animals and other research organisms

Policy information about studies involving animals; ARRIVE guidelines recommended for reporting animal research, and Sex and Gender in Research

Laboratory animals	Male Six-week-old wild-type C57Bl/6 mice.
Wild animals	The study did not involved wild animals.
Reporting on sex	The study did not used reporting on sex
Field-collected samples	Not applicable.
Ethics oversight	All experiments were conducted in accordance with the National Institutes of Health Guide for the Care and Use of Laboratory Animals and approved by the North China University of Science and Technology Animal Ethics Committee (No. 2021-SY-201).

Note that full information on the approval of the study protocol must also be provided in the manuscript.

Plants

Seed stocks	Not applicable.
Novel plant genotypes	Not applicable.
Authentication	Not applicable.

Flow Cytometry

Plots

Confirm that:

☒ The axis labels state the marker and fluorochrome used (e.g. CD4-FITC).

☒ The axis scales are clearly visible. Include numbers along axes only for bottom left plot of group (a 'group' is an analysis of identical markers).

☒ All plots are contour plots with outliers or pseudocolor plots.

☒ A numerical value for number of cells or percentage (with statistics) is provided.

Methodology

Sample preparation	Differently treated astrocytes were collected and incubated with MitoSOX , C11-BODIPY working solution for 30 min at 37°C. Post staining, the cells were washed three times and resuspended in PBS.
Instrument	C11-BODIPY, MitoSOX , and JC-1 were analysed using a flow Cytometer (CytExpert1.2; Beckman, USA)
Software	C11-BODIPY, MitoSOX , and JC-1 was analyzed using Flowjo 10.8.1 software.
Cell population abundance	Our study did not include the flow sorting experiment.
Gating strategy	C11-BODIPY and mtSOX analysis: First, perform quality control analysis on the cells with FSC-A as the X-axis and SSC A as the Y-axis to remove cell debris. Then, set singlet gates with FSC-H as the Y-axis and FSC-A as the X-axis, select the diagonal cell population, remove adherent cells, and determine single cells for analysis (A-B).<br>JC-1 analysis: The positive in the PE channel and the negative in the FITC channel was selected as the gating strategy (C).

☒ Tick this box to confirm that a figure exemplifying the gating strategy is provided in the Supplementary Information.
